# Supplementary material for: A fungal natural product that inhibits plant cellulose biosynthesis by disrupting cellulose synthase complexes
Source: Proc Natl Acad Sci U S A. 2026 Jun 9;123(24):e2602575123. doi: 10.1073/pnas.2602575123 (PMC13273347; doi:10.1073/pnas.2602575123)
Supplement: Supplementary file 1 — Appendix 01 (PDF) [file pnas.2602575123.sapp.pdf]

Supporting Information for

A Fungal Natural Product that Inhibits Plant Cellulose Biosynthesis by disrupting Cellulose Synthase Complexes

Zhongshou Wu[1,2,3#*], Lu Liu[1#], Wenyu Han[4#], Xingbo Cai[2,3], Pixian Xiao[1], Zuodong Sun[5], Chunsheng Yan[5], Silvana Reid[4], Yun Chen[1], Zhonghua Ma[1], Yi Tang[4,5*], Steven E. Jacobsen[2,3,6,7*]

*To whom correspondence may be addressed. Email: zhongshou6@zju.edu.cn; yitang@ucla.edu; jacobsen@ucla.edu;
#These authors contributed equally to this work

This PDF file includes:

Figures S1 to S9
Tables S1

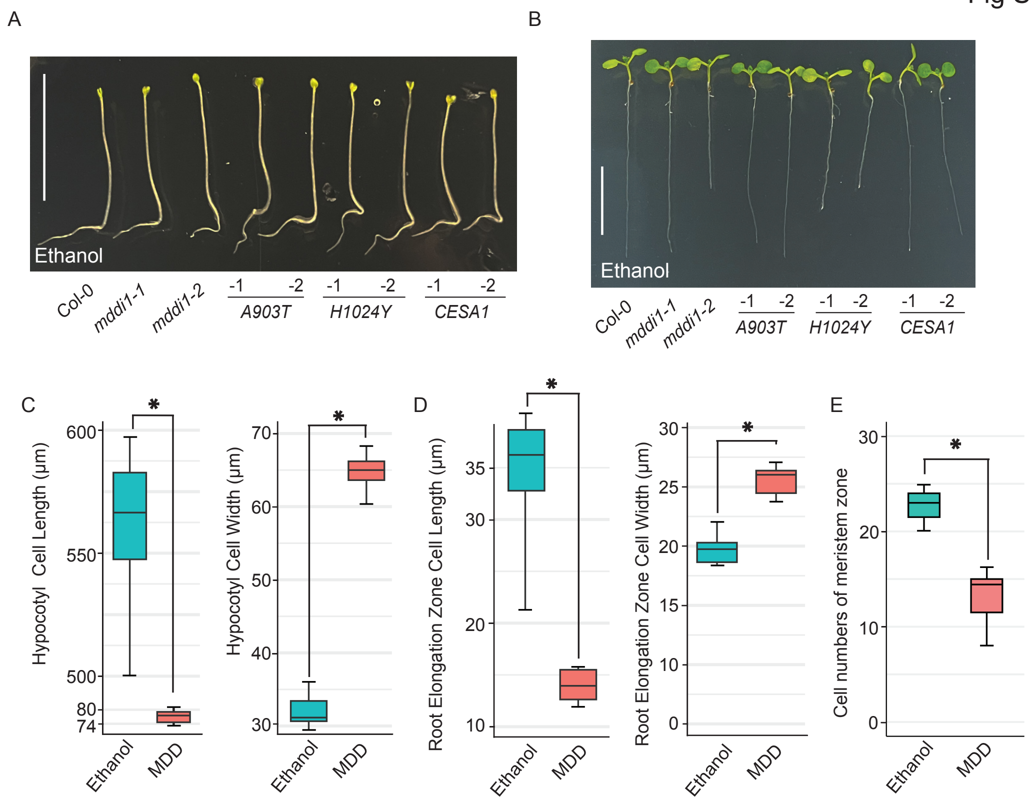

Fig. S1. MDD induces swelling in Arabidopsis hypocotyl and root cells.
A. Representative image of hypocotyl cells of 5-day-old dark-grown Arabidopsis seedlings grown on ½ MS medium supplemented with ethanol. Bars = 1.0 cm.
B. Representative image of root cells of 7-day-old light-grown Arabidopsis seedlings grown on ½ MS medium supplemented with ethanol. Bars = 1.0 cm.
C. Length and width of hypocotyl cells of 4-day-old dark-grown Arabidopsis seedlings grown on ½ MS medium supplemented with ethanol or 10 μM MDD. Statistical significance is indicated by different letters ($p < 0.01$). Error bars represent means ± standard deviations (n = 3).
D. Length and width of elongation zone root cells of 4-day-old light-grown Arabidopsis seedlings grown on ½ MS medium supplemented with ethanol or 10 μM MDD. Statistical significance is indicated by different letters ($p < 0.01$). Error bars represent means ± standard deviations (n = 3).
E. Cell numbers of meristem zone root cells of 4-day-old light-grown Arabidopsis seedlings grown on ½ MS medium supplemented with ethanol or 10 μM MDD. Statistical significance is indicated by different letters ($p < 0.01$). Error bars represent means ± standard deviations (n = 3).

Fig S2

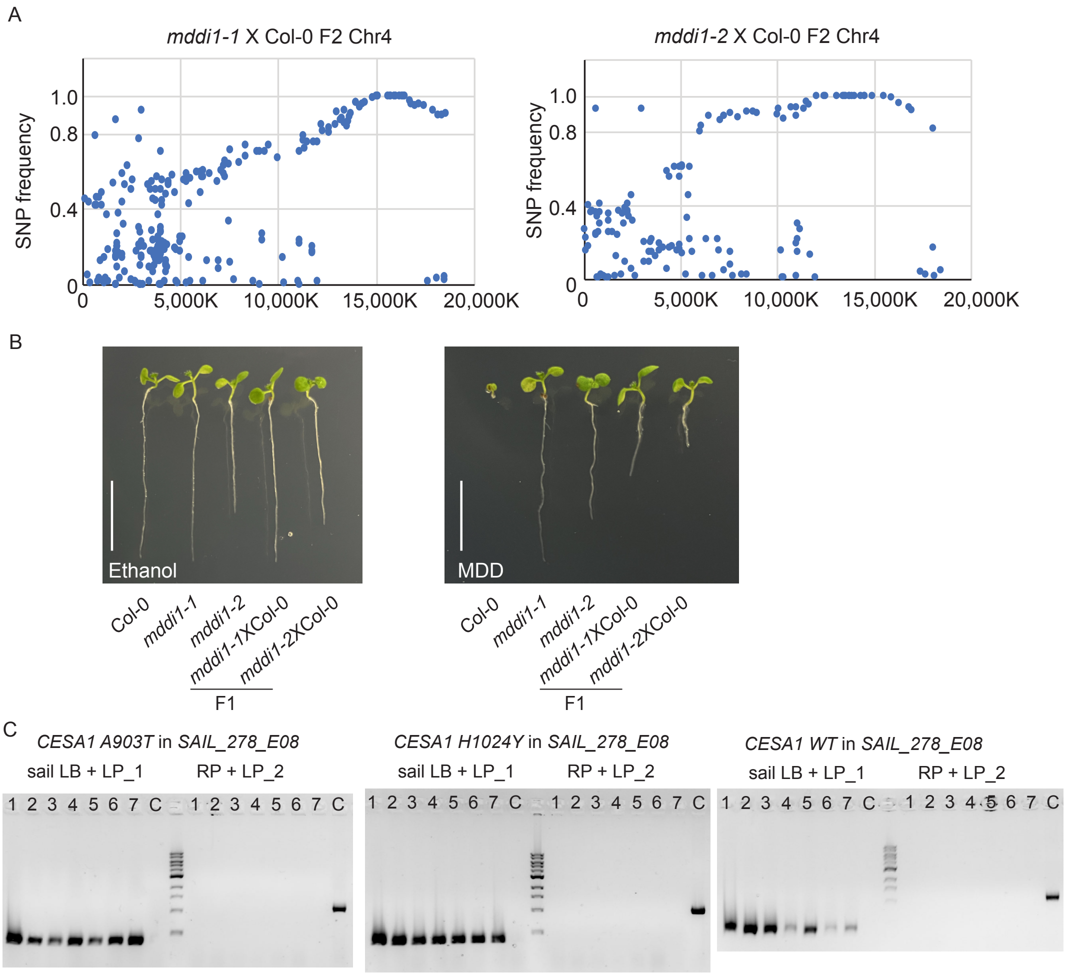

Fig. S2. Resistance to MDD is conferred by semi-dominant CESA1 alleles.
A. Dot plots of SNP frequency on chromosome 4 in *mddi1-1, mddi1-2* bulked backcross segregants.
B. Representative root image of *mddi1-1* and *mddi1-2* F1 backcrossed Arabidopsis seedlings grown on ½ MS medium supplemented with ethanol or 10 μM MDD. Bars = 1.0 cm.
C. Genotyping result showing the identification of *cesa1* null mutant when N-terminal GFP-tagged CESA1 A903T or CESA1 H1024Y or WT CESA1 were transformed into heterozygous *cesa1* knock-out mutants. The presence of T-DNA insertion in all T3 progenies from selected T2 lines confirmed homozygosity of the T-DNA insertion.

Fig S3

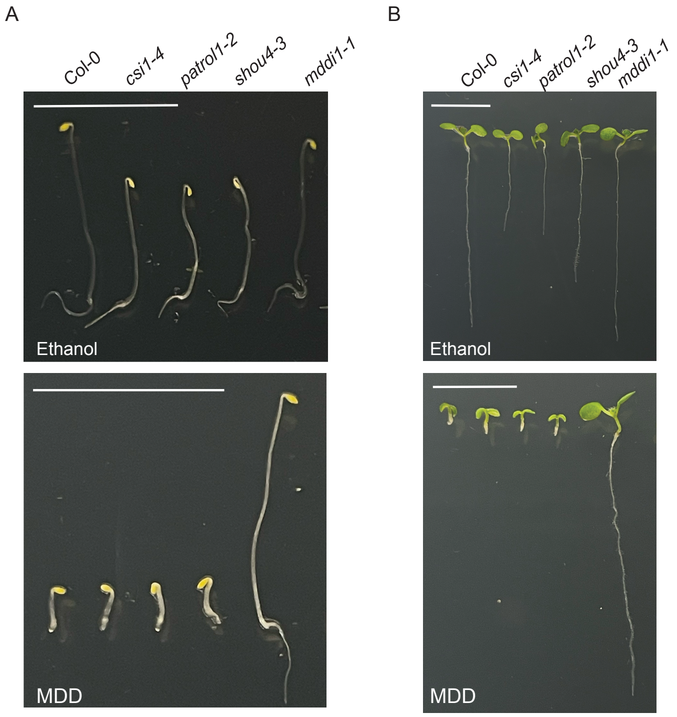

Fig. S3. MDD sensitivity remains unaltered by CSC complex quantity or regulatory mechanisms at the plasma membrane.
A. Representative image of 5-day-old dark-grown indicated Arabidopsis seedlings grown on ½ MS medium supplemented with ethanol or 10 μM MDD. Bars = 1.0 cm.
B. Representative image of 7-day-old light-grown indicated Arabidopsis seedlings grown on ½ MS medium supplemented with ethanol or 10 μM MDD. Bars = 1.0 cm.

Fig S4

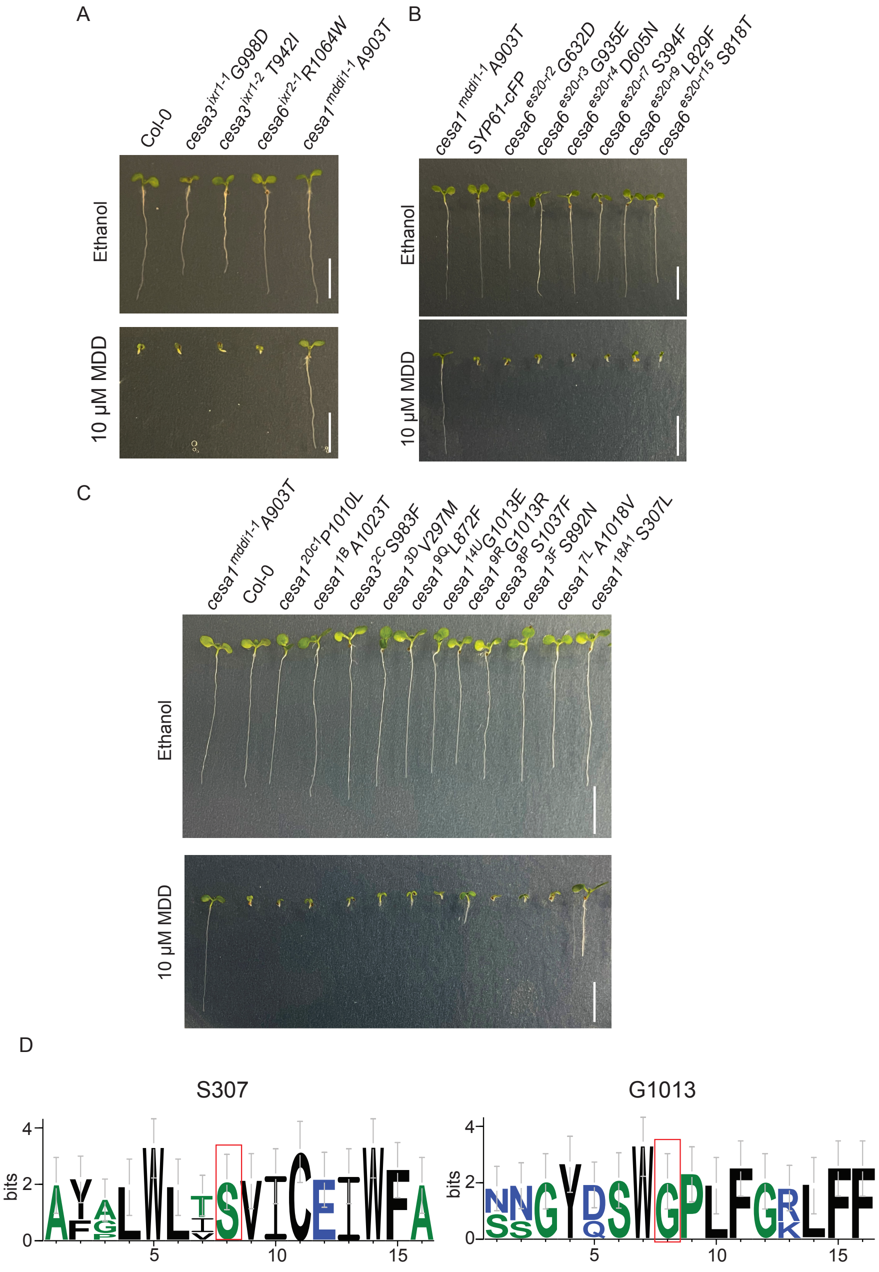

Fig. S4. Isoxaben, ES20-resistant mutants are susceptible to MDD.
A. Representative image of *cesa3*$^{ixr1-1}$ *G998D*, *cesa3*$^{ixr1-2}$ *T942I*, *cesa6*$^{ixr2-1}$ *R1064W* and *cesa1*$^{mddi1-1}$ *A903T* root growth of 7-day-old light-grown Arabidopsis seedlings grown on ½ MS medium supplemented with ethanol or 10 µM MDD. Bars = 1.0 cm.
B. Representative image of *cesa1*$^{mddi1-1}$ *A903T*, SYP61-CFP, *cesa6* $^{es20-r2}$ *G632D*, *cesa6* $^{es20-r3}$ *G935E*, *cesa6* $^{es20-r4}$ *D605N*, *cesa6* $^{es20-r7}$ *S394F*, *cesa6* $^{es20-r9}$ *L829F*, and *cesa6* $^{es20-r15}$ *S818T* root growth of 7-day-old light-grown Arabidopsis seedlings grown on ½ MS medium supplemented with ethanol or 10 µM MDD. Bars = 1.0 cm.
C. Representative image of *cesa1*$^{mddi1-1}$ *A903T*, Col-0, *cesa1*^{20c1} *P1010L*, *cesa1*^{1B} *A1023T*, *cesa3*^{2C} *S983F*, *cesa1*^{3D} *V297M*, *cesa1*^{9Q} *L872F*, *cesa1*^{14U} *G1013E*, *cesa1*^{9R} *G1013R*, *cesa3*^{8P} *S1037F*, *cesa1* ^{3F} *S892N*, *cesa1*^{7L} *A1018V*, and *cesa1*^{18A1} *S307L* root growth of 7-day-old light-

grown Arabidopsis seedlings grown on ½ MS medium supplemented with ethanol or 10 μM MDD. Bars = 1.0 cm.

D. Sequence logo assessment of residues in the additional mutation regions of primary cell wall CESA proteins illustrates the location and conservation of the mutated serine and glycine residues in CESA1.

Fig S5

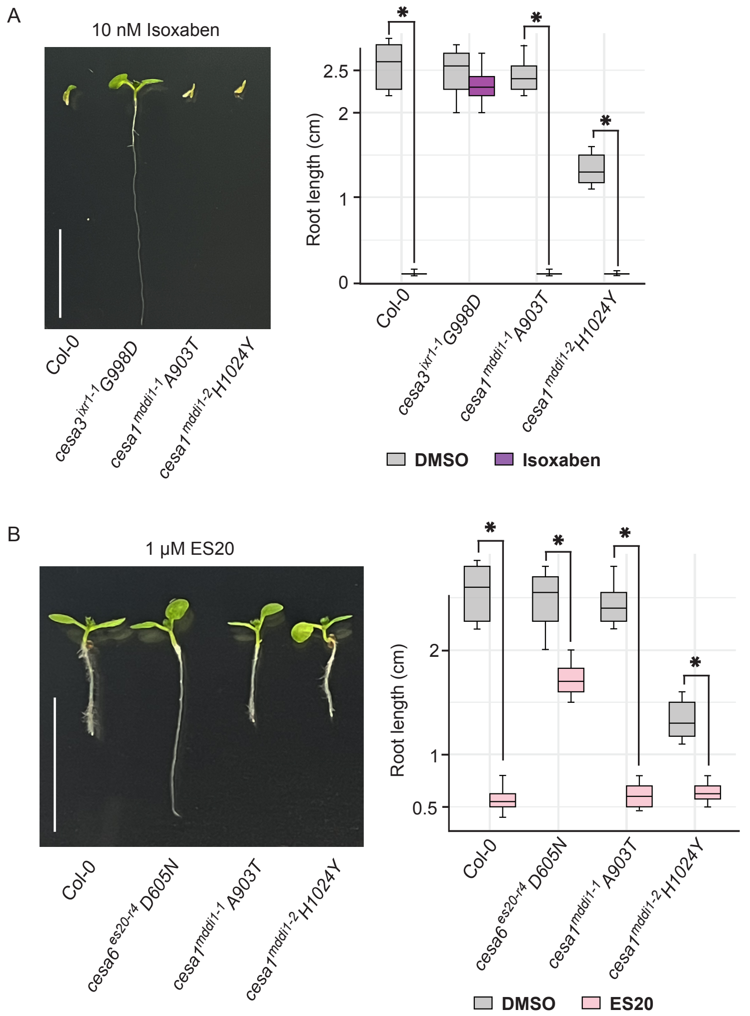

Fig. S5. *mddi* mutants are sensitive to isoxaben and ES20.

A. Representative image and quantification of Col-0, *cesa3*$^{ixr1\text{-}1}$ *G998D*, *cesa1*$^{mddi1\text{-}1}$ *A903T* and *cesa1*$^{mddi1\text{-}2}$ *H1024Y* root growth of 7-day-old light-grown Arabidopsis seedlings grown on ½ MS medium supplemented with 10 nM Isoxaben. Bars = 1.0 cm.

B. Representative image and quantification of Col-0, *cesa6*$^{es20\text{-}r4}$ *D605N*, *cesa1*$^{mddi1\text{-}1}$ *A903T* and *cesa1*$^{mddi1\text{-}2}$ *H1024Y* root growth of 7-day-old light-grown Arabidopsis seedlings grown on ½ MS medium supplemented with 1 μM ES20. Bars = 1.0 cm.

Fig S6

A

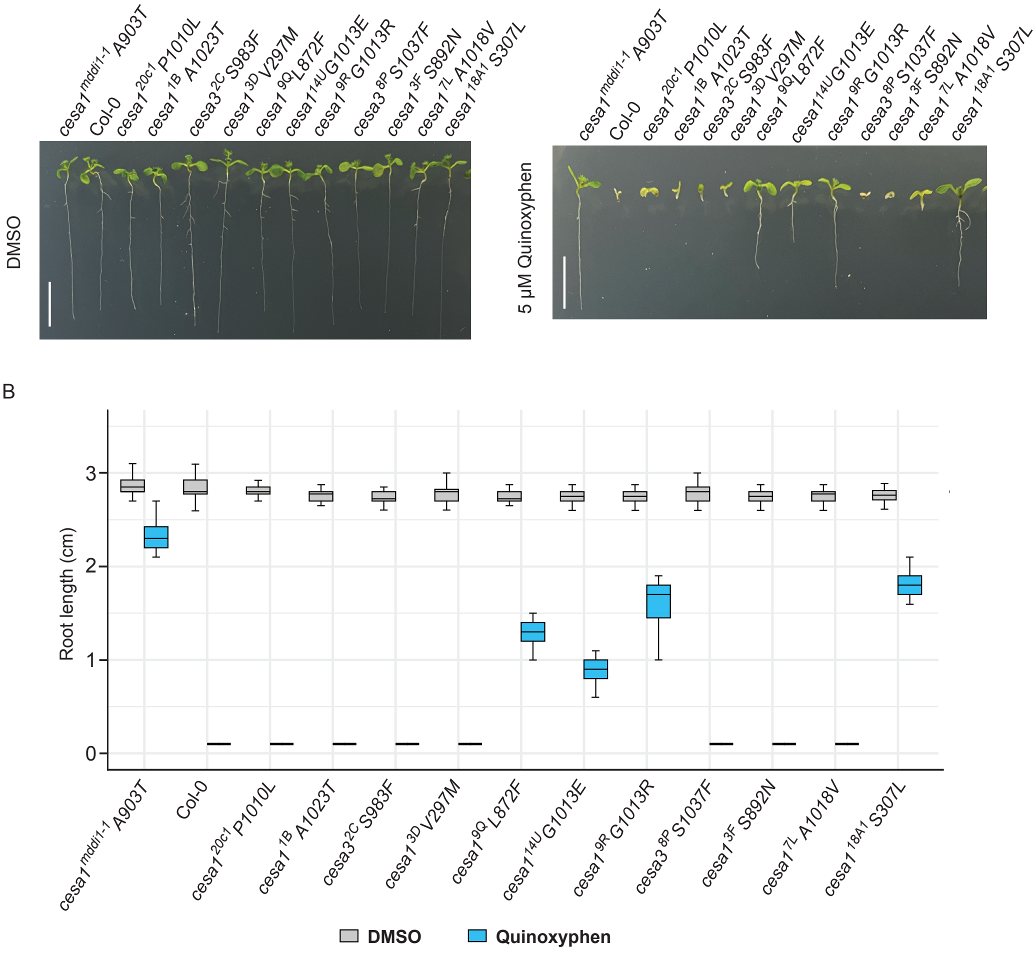

Fig. S6. C17 insensitive mutants showed partial resistant to quinoxyphen.
A-B. Representative image (A) and quantification (B) of $cesa1^{mddi1-1}$ *A903T*, Col-0, $cesa1^{20c1}$ *P1010L*, $cesa1^{1B}$ *A1023T*, $cesa3^{2C}$ *S983F*, $cesa1^{3D}$ *V297M*, $cesa1^{9Q}$ *L872F*, $cesa1^{14U}$ *G1013E*, $cesa1^{9R}$ *G1013R*, $cesa3^{8P}$ *S1037F*, $cesa1^{3F}$ *S892N*, $cesa1^{7L}$ *A1018V*, and $cesa1^{18A1}$ *S307L* root growth of 7-day-old light-grown Arabidopsis seedlings grown on ½ MS medium supplemented with DMSO or 5 μM Quinoxyphen. Bars = 1.0 cm.

Fig S7

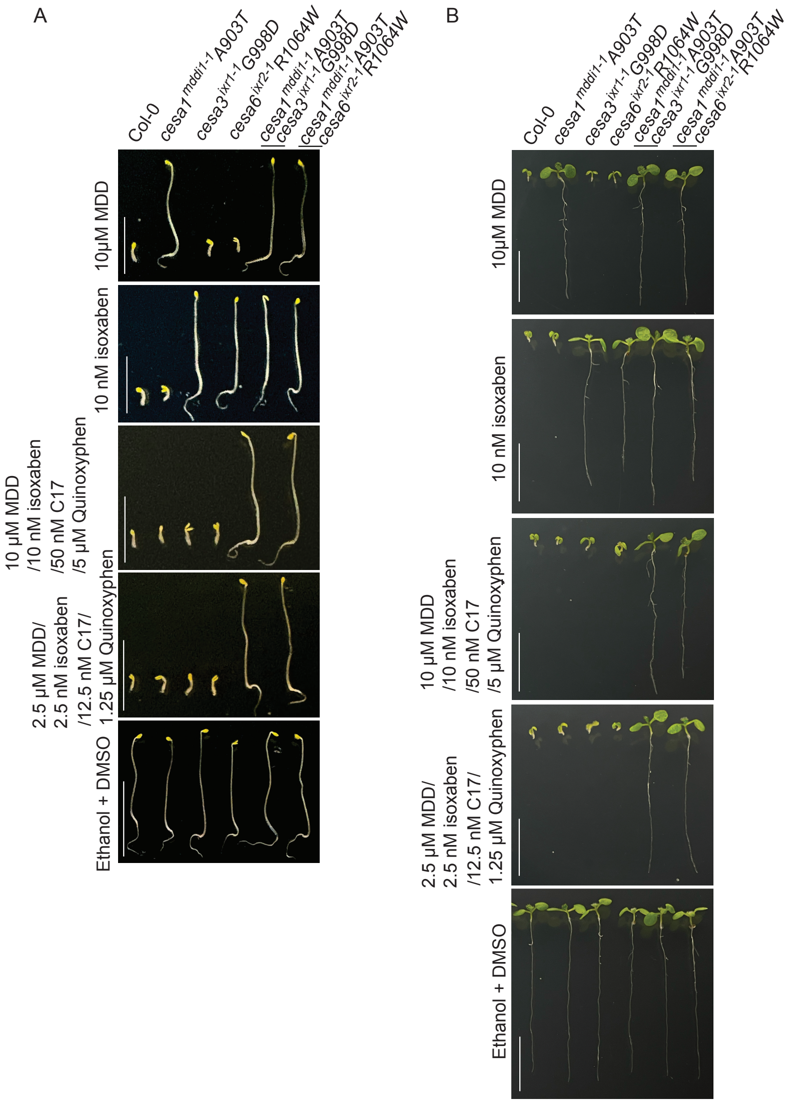

Fig. S7. Generation of MDD/isoxaben/C17/Quinoxyphen resistant mutants.
A. Representative image of Col-0, *cesa1*$^{mddi1-1}$ *A903T*, *cesa3*$^{ixr1-1}$ *G998D*, *cesa6*$^{ixr2-1}$ *R1064W*, *cesa1*$^{mddi1-1}$ *A903T cesa3*$^{ixr1-1}$ *G998D*, and *cesa1*$^{mddi1-1}$ *A903T cesa6*$^{ixr2-1}$ *R1064W* hypocotyl growth of 5-day-old dark-grown Arabidopsis seedlings grown on ½ MS medium supplemented with 10 µM MDD, 10 nM isoxaben, a combination treatment with 10 µM MDD/10 nM isoxaben/50 nM C17/5 µM Quinoxyphen, a combination treatment with 2.5 µM MDD/2.5 nM isoxaben/12.5 nM C17/1.25 µM Quinoxyphen. Bars = 1.0 cm.
B. Representative image of Col-0, *cesa1*$^{mddi1-1}$ *A903T*, *cesa3*$^{ixr1-1}$ *G998D*, *cesa6*$^{ixr2-1}$ *R1064W*, *cesa1*$^{mddi1-1}$ *A903T cesa3*$^{ixr1-1}$ *G998D*, and *cesa1*$^{mddi1-1}$ *A903T cesa6*$^{ixr2-1}$ *R1064W* root growth of 7-day-old light-grown Arabidopsis seedlings grown on ½ MS medium supplemented with 10 µM MDD, 10 nM isoxaben, a combination treatment with 10 µM MDD/10 nM isoxaben/50 nM C17/5 µM Quinoxyphen, or a combination treatment with 2.5 µM MDD/2.5 nM isoxaben/12.5 nM C17/1.25 µM Quinoxyphen. Bars = 1.0 cm.

Fig S8

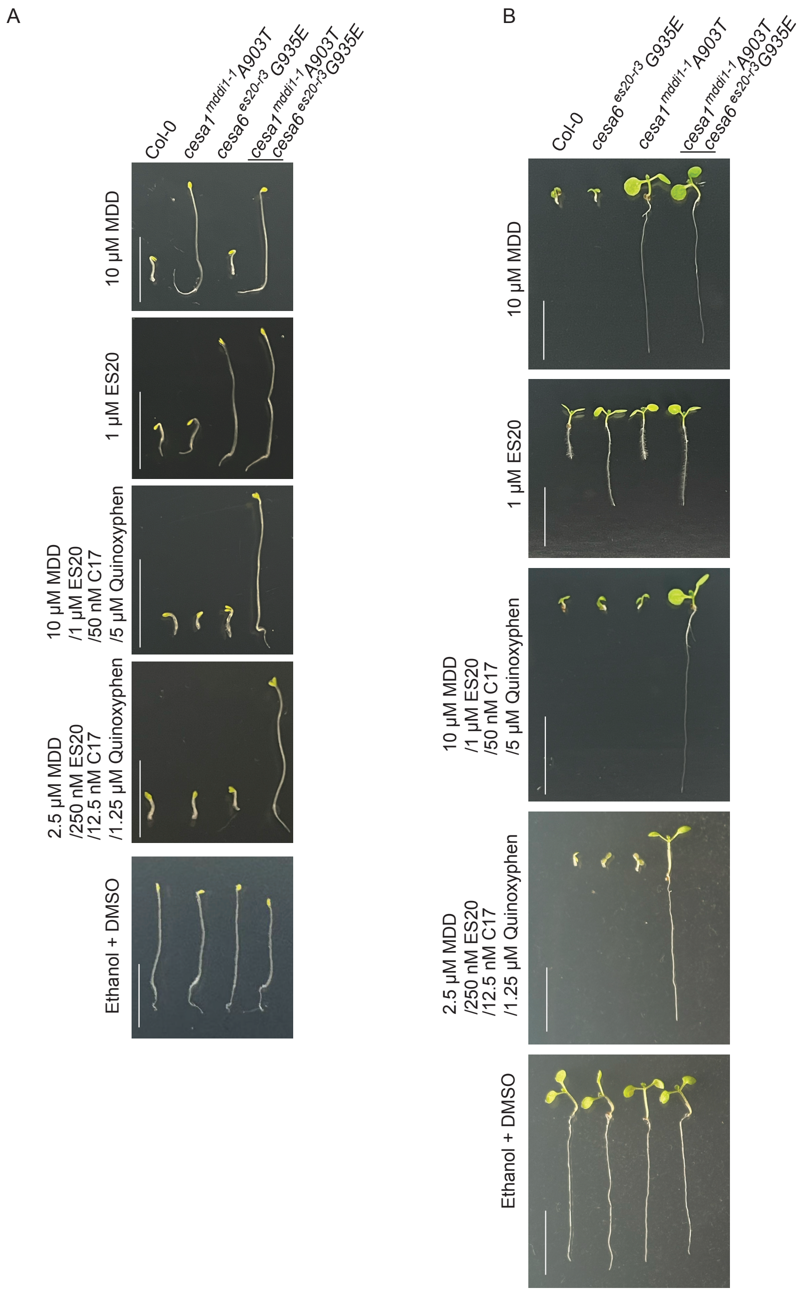

Fig. S8. Generation of MDD/ES20/C17/Quinoxyphen resistant mutants.

A. Representative image of Col-0, *cesa1*$^{mddi1\text{-}1}$ *A903T*, *cesa6*$^{es20\text{-}r3}$ *G935E*, and *cesa1*$^{mddi1\text{-}1}$ *A903T cesa6*$^{es20\text{-}r3}$ *G935E* hypocotyl growth of 5-day-old dark-grown Arabidopsis seedlings grown on ½ MS medium supplemented with 10 µM MDD, 1 µM ES20, a combination treatment with 10 µM MDD/1 µM ES20/50 nM C17/5 µM Quinoxyphen, a combination treatment with 2.5 µM MDD/250 nM ES20/12.5 nM C17/1.25 µM Quinoxyphen. Bars = 1.0 cm.

B. Representative image of Col-0, *cesa1*$^{mddi1\text{-}1}$ *A903T*, *cesa6*$^{es20\text{-}r3}$ *G935E*, and *cesa1*$^{mddi1\text{-}1}$ *A903T cesa6*$^{es20\text{-}r3}$ *G935E* root growth of 7-day-old light-grown Arabidopsis seedlings grown on ½ MS medium supplemented with 10 µM MDD, 1 µM ES20, a combination treatment with 10 µM

MDD/1 μM ES20/50 nM C17/5 μM Quinoxyphen, a combination treatment with 2.5 μM MDD/250 nM ES20/12.5 nM C17/1.25 μM Quinoxyphen. Bars = 1.0 cm.

Supplemental Table1. Primers used in this study

Primer name	primer sequence
SAIL_278_E08_LP_1	CCAGAACTGCTCGTTCCTCC
SAIL_278_E08_LP_2	AGCTAATCTTGGACATGGAA
SAIL_278_E08 _RP	CCTGCGTTCAAGGGATCTGC
CESA1-F1	CCGCGGCCGCCCCCTTCACCCGAAGTCGGATTCGACATCC
CESA1-R1	CTCCTCGCCCTTGCTCACCATCGCAGCCACCGACACACAGA
CESA1-F2	TCTGTGTGTCGGTGGCTGCGATGGTGAGCAAGGGCGAGGAG
CESA1-R2	AAGCCGGCACTGGCCTCCATAGCCTTGTACAGCTCGTCCA
CESA1-F3	TGGACGAGCTGTACAAGGCTATGGAGGCCAGTGCCGGCTT
CESA1-R3	GGGTCGGCGCGCCCACCCTTCTAAAAGACACCTCCTTTGC
ixr1_F	AATGGTGGAGAAACGAGCAG
ixr1_R	CAACAGTTGATTCCACATTC
es20r3_F	TGCGAGTATCCTCTTCATGG
es20r3_R	GGGATGAGAAGTGAAGTCCA
ixr2_F	AGACTGTTCTTTGCACTTTG
ixr2_R	TCACAAGCAGTCTAAACCAC
mddi1-F	ACATTGTCCTATCTGGTATG
mddi1-R	AGTAGACCTTGGAAGACAGC
